# Mouse Models for Filovirus Infections

**DOI:** 10.3390/v4091477

**Published:** 2012-09-07

**Authors:** Steven B. Bradfute, Kelly L. Warfield, Mike Bray

**Affiliations:** 1 Molecular Genetics and Microbiology, University of New Mexico, Albuquerque, NM 87131, USA; 2 Vaccine Development, Integrated Biotherapeutics, Inc., Gaithersburg, MD 20878, USA; Email: kelly@integratedbiotherapeutics.com; 3 Division of Clinical Research, National Institute of Allergy and Infectious Diseases, National Institutes of Health, Bethesda, MD 20892, USA; Email: MBray@niaid.nih.gov

**Keywords:** filovirus, Ebola, Marburg, mouse models, hemorrhagic fever

## Abstract

The filoviruses marburg- and ebolaviruses can cause severe hemorrhagic fever (HF) in humans and nonhuman primates. Because many cases have occurred in geographical areas lacking a medical research infrastructure, most studies of the pathogenesis of filoviral HF, and all efforts to develop drugs and vaccines, have been carried out in biocontainment laboratories in non-endemic countries, using nonhuman primates (NHPs), guinea pigs and mice as animal models. NHPs appear to closely mirror filoviral HF in humans (based on limited clinical data), but only small numbers may be used in carefully regulated experiments; much research is therefore done in rodents. Because of their availability in large numbers and the existence of a wealth of reagents for biochemical and immunological testing, mice have become the preferred small animal model for filovirus research. Since the first experiments following the initial 1967 marburgvirus outbreak, wild-type or mouse-adapted viruses have been tested in immunocompetent or immunodeficient mice. In this paper, we review how these types of studies have been used to investigate the pathogenesis of filoviral disease, identify immune responses to infection and evaluate antiviral drugs and vaccines. We also discuss the strengths and weaknesses of murine models for filovirus research, and identify important questions for further study.

## 1. Introduction

The filoviruses are negative-sense, single-stranded enveloped RNA viruses that can cause severe hemorrhagic fever (HF) in humans and nonhuman primates (NHPs) (reviewed in [1]). The family *Filoviridae* is divided into three genera: ebolaviruses, marburgviruses, and cuevaviruses [2,3,4]. Of the five ebolavirus species, three are highly pathogenic for humans: Ebola (EBOV), formerly known as Zaire ebolavirus, with case fatality rates (CFR) in African epidemics ranging from 70%–90%; Sudan (SUDV), with an average CFR of 50%; and the recently identified Bundibugyo virus (BDBV), which caused fatal disease in about 25% of patients in the only known outbreak. The Reston virus (RESTV) has never been known to cause recognized disease in humans, and the only person known to have been infected with the Tai Forest virus (TAFV) survived. There are two marburgviruses, Marburg (MARV) and Ravn (RAVV), which are as lethal as EBOV for humans [5]. The newly described cuevavirus (Lloviu) was discovered during an investigation of a die-off of bats in Spain, and was discovered by genetic sequencing; its virulence for humans or NHPs has not yet been assessed. At this time, isolation of infectious cuevavirus has not been reported.

The filoviruses were first recognized as the cause of human disease during an outbreak of severe HF in Marburg, Germany in 1967. Since that time, about 2,000 confirmed cases of filoviral disease have been identified, almost all in African countries with a limited medical infrastructure. As a consequence, most research on the pathogenesis of ebolaviruses and marburgviruses, and evaluations of potential antiviral drugs and vaccines, have been performed in biocontainment laboratories. Several filovirus animal models have been developed, including NHPs, guinea pigs, hamsters and mice. NHPs succumb when challenged with all strains of ebolaviruses and marburgvirus, and the disease appears to closely mirror what is known of filovirus disease of humans, making them excellent models for research, although there are differences in filovirus pathogenesis depending on the NHP species tested. However, because these animals are expensive and can only be used in small numbers, most preliminary studies of filovirus infection are performed in rodents. Additionally, not all BSL-4 laboratories are equipped to house NHPs. Guinea pigs have been used for research since the initial marburgvirus outbreak in 1967, but because of their comparatively large size and the lack of immunological reagents and test kits, fewer studies are performed in these animals. Furthermore, transgenic or knockout animals are not available in the NHP or guinea pig models, making mechanistic studies difficult. The majority of current small animal research is therefore performed in mice. 

Soon after the first recognized outbreak of marburgvirus disease (MVD) in 1967 and of ebolavirus disease (EVD) in 1976, investigators found that viruses isolated from patients caused lethal infection in newborn mice, when inoculated by the intracerebral (i.c.) or intraperitoneal (i.p.) route [6,7,8]. However, because newborn mice cannot be used to effectively study disease pathogenesis or evaluate vaccines, and have limited value for antiviral drug testing, more recent efforts have focused on developing models of filoviral disease in adult mice. Such studies can be divided into three types: those in which immunocompetent mice are inoculated with filoviruses recovered from human patients or nonhuman primates (“wild-type viruses”); those in which immunocompetent mice are inoculated with wild-type viruses that have been “adapted” to virulence through sequential passage (“mouse‑adapted viruses”); and those in which mice with defective innate or adaptive immune responses are inoculated with wild‑type or mouse-adapted viruses. In this paper, we first briefly summarize the pathogenesis of filoviral disease, as it is currently understood, then review how the above three approaches have been used to produce models of filoviral infection in mice, noting the principal pathologic findings and comparing them to those in NHPs. We then summarize how mouse models have been used to evaluate antiviral drugs and vaccines. In the concluding section, we discuss the strengths and weaknesses of murine models for filovirus research, and identify important questions for further study. 

## 2. Pathogenesis of Filovirus Disease in Humans and NHPs

A primary question in filovirus research is whether rodent models accurately mirror the features of ebolavirus and marburgvirus pathogenesis in humans. It is important to note that clinical data on human filovirus infections are limited. Before reviewing the features of infection of mice, it is therefore necessary to summarize the pathogenesis of filovirus disease, as it has been elucidated from limited clinical studies of patients in the 1967 marburgvirus outbreak and subsequent epidemics in Africa, and from time-course experiments in NHPs. While the exact mechanism of filovirus disease are unknown, one view suggests that blocking of type I interferon (IFN) responses in conjunction with a dysregulated pro-inflammatory cytokine response leads to disease. Aided by viral proteins, VP24 and VP35 (or VP40 for MARV) that block type I IFN responses, filoviruses replicate to high titers and disseminate rapidly in the blood and lymph, infecting a wide range of cells throughout the body and generating a high viremia that in fatal cases persists through death. At the same time, the release of large quantities of proinflammatory, vasoactive mediators from infected macrophages and other cells produces a cytokine “storm”, resulting in a diffuse increase in vascular permeability that leads to a fall in plasma volume, hypotension, multi-organ failure and shock. It has been suggested that the synthesis by macrophages of cell-surface tissue factor (TF) triggers the extrinsic coagulation system, leading to disseminated intravascular coagulation (DIC) [9]. Activation of coagulation is visible microscopically, in the form of fibrin deposition at foci of viral replication in the spleen and other tissues. However, the role of coagulopathy in lethal infection is unknown, since DIC may not be generated in certain NHP infections, and different NHP species have differing coagulation and fibrin deposition outcomes after infection. 

A characteristic pattern of blood cell counts occurs over the course of illness, with an initial increase in immature granulocytes, profound thrombocytopenia, and an initial decline in the lymphocyte count that is often followed by a sharp rebound during the 1–2 days before death. Because filoviruses are able to replicate in a wide variety of cell types, viremia leads to massive infection and necrosis of parenchymal cells of the liver, adrenal glands and other organs. Liver and kidney function is diminished, as measured by increased tissue enzymes and molecules in the blood. Filovirus disease therefore likely results from a combination of severe systemic inflammation and massive tissue damage. 

## 3. Filovirus Infection of Mice

### 3.1. Wild-Type Filovirus Infections of Wild-Type Mice

Soon after the first outbreaks of MARV and EBOV infections, researchers found that viruses isolated from patients caused lethal illness in newborn mice when inoculated by the i.c. or i.p. route [7,8,10,11]. Because filovirus plaquing methods in tissue culture had not yet been developed, newborn mice were used to measure the content of infectious virus in serum and other preparations. The method was eventually found to be more sensitive than titrations in tissue culture, capable of detecting less than 1 plaque-forming unit (pfu) of virus [12]. However, because of their small size and immature immune systems, newborn mice could not be used for pathogenesis studies or to test candidate vaccines. 

Adult immunocompetent mice are solidly resistant to wild-type filoviruses inoculated by any route, although the viruses replicate before being cleared [13,14]. Infection of knockout or transgenic adult mice can therefore be used to identify immunologic mechanisms required for resistance to infection. 

### 3.2. Adaptation of Filoviruses to Virulence for Mice through Sequential Passage

#### 3.2.1. EBOV

The 1976 Mayinga isolate of EBOV was adapted to lethal virulence for adult, immunocompetent mice through sequential passage in newborn, suckling and progressively older weanling mice, using i.p. inoculation of suspensions of liver homogenates [15]. The resultant plaque-purified “mouse-adapted” ebolavirus (maEBOV) was uniformly lethal for adult BALB/c, CD-1, or C57BL/6 mice when inoculated i.p., with an LD_50_ of approximately 0.03 pfu, which was shown by electron microscopy to be equivalent to a single virion. Remarkably, the inoculation of the same virus by the subcutaneous (s.c.) or intramuscular (i.m.) route did not produce visible illness, even in doses of 10^6^ pfu s.c. [15]. 

Sequencing showed that maEBOV contains 8 amino acid changes, compared to the original wild-type virus [16]. By constructing recombinant viruses containing genes of maEBOV and wild-type virus (wtEBOV), the determinants of virulence were localized to the nucleoprotein (NP) and VP24 genes. Recombinant wild-type viruses containing mouse NP and VP24 genes were lethal in mice and resistant to the effects of type I IFN in an *in vitro* assay [16]. As noted below, the EBOV VP24 and VP35 proteins have been shown to block type I IFN responses through a variety of mechanisms [17]; however, VP35 does not play a role in the virulence of maEBOV for mice.

**Figure 1:**
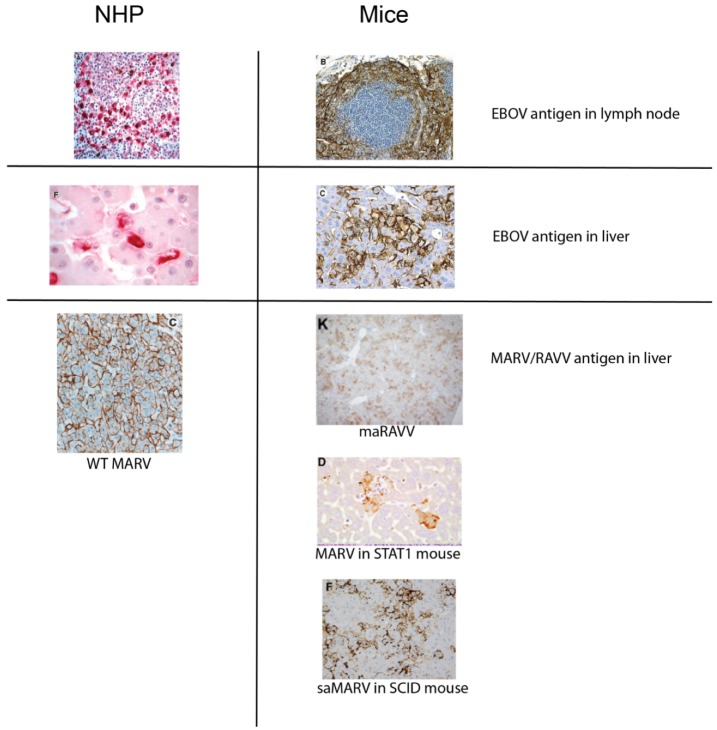
Filovirus antigen staining in nonhuman primates (NHPs) (left) and mice (right) lethally infected with Ebola (EBOV), Marburg (MARV), or Ravn (RAVV). Images adapted from references [14,18,21,31,32,33].

**Figure 2:**
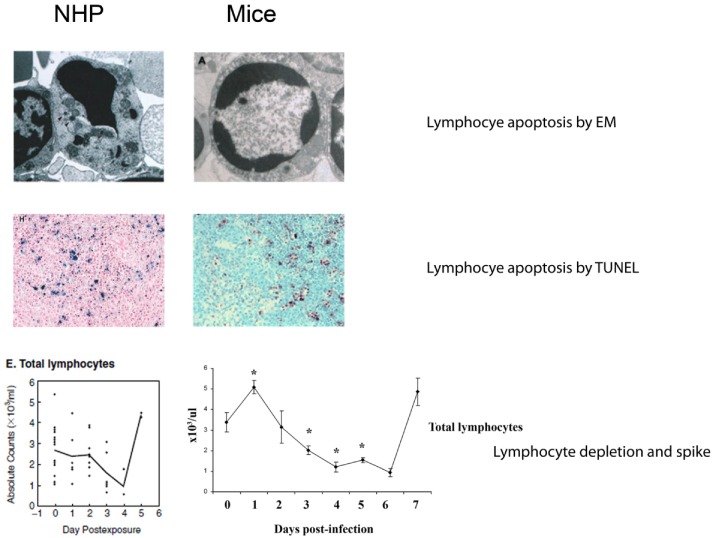
Lymphocyte responses in fatal EBOV infection in NHPs (left) and mice (right). Figures adapted from references [19,21,22,36,37]. Right middle panel, Copyright 2010. The American Association of Immunologists, Inc. Right bottom panel, Copyright 2008. The American Association of Immunologists, Inc.

The disease produced in adult, immunocompetent mice by i.p. inoculation of maEBOV resembles EBOV infection of NHPs in many respects. The virus replicates rapidly, reaching serum titers of up to 10^9^ pfu/mL. As seen in NHPs, the systemic spread of virus (Figure 1) results in extensive infection and necrosis of the liver, spleen and other organs [15]. Histopathological and biochemical data show that liver and kidney function is diminished in mice, similar to that seen in NHPs [18]. Widespread lymphocyte apoptosis, a hallmark of lethal EBOV infection in humans and NHPs, is observed in mice infected with maEBOV [9,15,18,19,20,21,22,23,24,25] (Figure 2). The pattern of proinflammatory cytokine production, including tumor necrosis factor (TNF)-α, IFN-γ, IL-8, MIP-1α, MIP-1β, and MCP-1 also resembles that seen in EBOV-infected NHPs [25,26,27,28,29,30]. Lymphocyte activation, as determined by lymphoblast formation, increased T-cell CD44 expression, and an increase in lymphocyte number in the blood late in infection, is found in mouse and NHP EBOV models. Therefore, mice clearly have similar immune responses to EBOV infection compared to NHPs. However, it should be noted that in-depth immune response studies have not been published for any filovirus model; further study may reveal differences. 

Although fatal infection of mice by maEBOV resembles EBOV disease in NHPs in many respects, certain differences are observed. As noted, mice are only sensitive to virus inoculated i.p., but not s.c. or i.m., while NHPs are susceptible to infection by small doses of virus by these routes [15]. Fibrin deposition and breakdown, resulting in the appearance of D-dimers in the plasma and fibrin deposition at sites of viral replication in the spleen and other tissues, are seen in certain (but not all) filovirus‑infected NHPs. In mice, by contrast, infection with maEBOV does not result in visible fibrin deposition in tissue sections, although D-dimer levels have not been tested [15,21]. However, mice develop a marked thrombocytopenia, a hallmark of HF, and D-dimers have been detected in mice infected with maRAVV (see below), suggesting that murine models may be more relevant for the study of filovirus-induced coagulopathy than originally thought. Treatment with the anti-coagulants recombinant nematode anticoagulant protein C2 (rNAPC2) or activated protein C (that, notably, affect other systems, such as anti-inflammatory actions), improves the outcome of illness in macaques [31,34,35]. Anti-coagulant treatment of maEBOV-infected mice could experimentally show whether coagulopathy is a significant factor in the disease observed in the mouse models. 

#### 3.2.2. Marburgviruses

Variants of MARV and RAVV that are virulent for adult, immunocompetent mice have been isolated through sequential passage in severe, combined immunodeficient (SCID) mice [33]. As discussed below, SCID mice, which lack B and T cells, but have intact IFN responses, develop a lengthy illness when inoculated with wild-type filoviruses, dying 3–4 weeks after infection [33]. However, after a small number of animal-to-animal passages, both RAVV and the Ci67 and Musoke strains of MARV caused rapidly lethal disease. These SCID-adapted viruses were used to demonstrate protection by antibodies or antisense therapeutics [33]. The SCID-adapted RAVV was then passaged further in normal adult mice, generating a lethal mouse-adapted variant (maRAVV) which caused death in 5–10 days after infection [14]. 

The disease produced in maRAVV-infected immunocompetent adult mice resembles RAVV infection of guinea pigs and NHPs in a number of ways, including high viremia and viral tissue titers, induction of D-dimers (fibrin degradation products), platelet loss, profound loss of circulating and tissue lymphocytes, and marked liver damage [14]. Similar to the maEBOV mouse model, only i.p. injection of mice caused lethal disease among the routes tested (i.n., s.c., footpad, or i.m.) at either 1,000 or 100,000 pfu challenge doses [14]. This mouse-adapted RAVV can now be used in a similar manner to the maEBOV mouse model to assist in development of novel vaccines, therapeutics and understanding of the virulence factors associated with RAVV infection. A mouse-adapted MARV has also been reported [38]. This maMARV appears to be less virulent in mice than maRAVV, although both viruses replicate to fairly similar levels in multiple organs (including spleen, lymph nodes, kidney, and gonads) [38].

### 3.3. Wild-Type Filovirus Infection of Immunodeficient Mice

#### 3.3.1. Mice with Defective Innate Antiviral Responses

Knockout mice lacking the receptor for interferon-α and β (IFN-α/βR−/−) or the cytoplasmic Signal Transducer and Activator of Transcription-1 (STAT1) protein are susceptible to infection by a wide range of filoviruses that do not cause disease in wild-type mice. Both types of mice succumb to infection when inoculated with wild-type EBOV, MARV and RAVV [13,32,39,40]. Interestingly, EBOV-Mayinga was lethal but EBOV-Kikwit was not [13,39], suggesting the possibility of variation in type I IFN responses to different isolates of the same virus. Likewise, different isolates of SUDV appeared to vary in their lethality in type I IFN deficient mice [13,39]. Additionally, wild-type mice treated with neutralizing antibody against IFN-alpha/beta are susceptible to infection with some wild‑type strains of ebolavirus [13]. Interestingly, TAFV, which was not lethal in the only known human case, and RESTV, which has not caused disease in humans, were the least virulent filoviruses for type I IFN mutant mice, although one study showed strain-dependent lethality after RESTV infection [40]. In contrast to immunocompetent mice, which become ill only when maEBOV or maRAVV was inoculated i.p., knockout mice lacking effective type I IFN responses are susceptible to infection by other routes, including aerosol exposure [39]. Together, these results suggest that viral inhibition of type I IFN responses is critical for pathogenesis of certain filovirus infections. 

#### 3.3.2. Mice with Defective Adaptive Immune Responses

SCID mice, which lack B and T cells, can be lethally infected with wild-type filoviruses (EBOV, SUDV, RAVV, and MARV) although the time to death in these mice is greatly extended relative to other lethal mouse models [13,33]. Warfield *et al.* [33] tried a different approach by serially passaging liver homogenates from wild-type MARV or RAVV-infected SCID mice into recipient SCID mice. Three to ten passages, depending on the MARV or RAVV used (Ci67, Ravn, or Musoke), generated rapidly lethal SCID-adapted viruses. These SCID-adapted MARV or RAVV models were useful platforms for therapeutic testing, showing protection or delayed time to death mediated by antibodies or antisense therapeutics [33]. 

## 4. Studies of Host Responses to Filovirus Infection in Mice

### 4.1. Type I IFN Responses

The mouse model of maEBOV infection has proven to be extremely useful in exploring the requirement of type I interferon in resistance to filovirus infection. Drug studies have shown an interesting link between IFN signaling and filovirus infection. Mice treated with 3-deazaneplanocin A were completely protected from maEBOV infection, but this protection was abolished when mice were injected with neutralizing antibodies against IFN-alpha/beta [13]. Further work showed that treatment with the drug increased IFN-alpha levels in infected mice, suggesting that 3-deazaneplanocin A, which inhibits cellular methylation reactions, may exert its antiviral effect both by restricting cap methylation of viral mRNA and by inducing a heightened, early type I IFN response [41,42]. Indeed, treatment of NHPs with IFN-alpha2b has been shown to prolong time-to-death and resulted in delayed viremia [43]. However, a preliminary study using multiple doses of 3-deazanoplanocin A did not protect NHPs from EBOV infection, nor did it increase type I IFN expression [41]. Further work on this topic could be relevant to understanding filovirus pathogenesis.

### 4.2. Determinants of Filovirus Virulence

The development of maEBOV provided an opportunity to compare the sequences of viral variants and determine the importance of individual genes for lethal infection. maEBOV contains eight amino acid changes compared to wtEBOV: one change in each NP, VP35, and VP24, 3 in GP, and two in L. In addition, there are 2 nucleotide mutations in the non-coding regions of VP30 and VP24 [16]. In an elegant study, Ebihara *et al.* [16] substituted genes from wtEBOV into maEBOV, or vice-versa, and infected mice with these recombinant viruses. By calculating the LD50 of the different variants, they were able to determine which mutations were important for adaptation to mice. These studies revealed that substitution of both mouse-adapted NP and VP24 into wtEBOV was sufficient to cause lethality in mice, although substitution of only one of the genes was not sufficient. In a reciprocal experiment, substitution of either wild-type NP or wild-type VP24 into maEBOV abrogated its lethality. Wild-type virus carrying mouse-adapted NP and VP24 was resistant to the effects of type I IFN in an *in vitro* viral replication assay, suggesting that the mouse-adapted virus mutations may inhibit IFN activity. This correlates with the requirement of intact IFN signaling in mice for resistance to wild-type filovirus infection, as discussed above. Additionally, EBOV VP24 and VP35, and RAVV VP40, have been shown to inhibit type I IFN responses in host cells via a variety of mechanisms (reviewed in [17]). maRAVV and maMARV contain mutations in VP40, emphasizing the putative importance of this gene in marburgvirus pathogenesis [38]. Interestingly, large doses of maEBOV (10^6^ pfu) are not lethal in mice, and it has been speculated that this could be due to massive activation of innate immune responses [16].

### 4.3. Basis of Inherent Resistance to Infection

The resistance of adult immunocompetent mice to wild-type filoviruses inoculated by any route and to maEBOV and maRAVV inoculated by routes other than i.p. makes it possible to use mice as a tool to study the basis of successful resistance to infection. 

In mice challenged with wild-type RAVV, no change in white blood cell count, lymphocyte percentage, or lymphocyte number was observed, although these parameters dropped in maRAVV infected mice [14]. Mice infected with the wild-type virus had a drop in blood B cell numbers compared to uninfected mice, but did not show an increase in CD4^+^ or CD8^+^ T cells. maRAVV-infected mice demonstrated a decrease in circulating B, NK, and CD4^+^ and CD8^+^ T cell numbers compared to wtRAVV-infected mice. Many cytokines and chemokines were increased in the sera of maRAVV-infected mice relative to wtRAVV-infected mice [14]. 

The basis of protection has also been characterized in normal mice challenged s.c. with maEBOV or wtRAVV [14,15]. Mice “immunized” by subcutaneous maEBOV challenge have increased IFN-α and IFN-γ levels after i.p. re-challenge, compared to controls [44]. Use of knockout mice lacking various lymphocyte subsets demonstrated that CD8^+^ T cells, but not B cells or CD4^+^ T cells, were required for protection against s.c. maEBOV [45]. This CD8^+^ T cell-mediated protection is likely perforin-dependent, as KO mice lacking perforin succumb to s.c. infection, but Fas- or IFN-γ-KO mice were resistant and cleared the infection [45].

One study used wtRAVV infection of mice to identify CD8^+^ T cell epitopes that were generated during nonlethal infection [46]. These peptides were then used to expand CD8^+^ T cells, which were adoptively transferred to mice infected with maRAVV to analyze their protective capability. Furthermore, the cytokine secreting and cytolytic capabilities of protective and non-protective T cell clones were analyzed to begin to study mechanisms of immune responses and correlates of immunity [46]. The use of humanized mice may expand these studies to analyze epitope mapping of human responses to filovirus infection; indeed, certain MHC II haplotypes in humans are correlated with protection from SUDV [47].

### 4.4. Lymphocyte Apoptosis

Rather than being unique to filoviral infection, a decrease in circulating lymphocytes and marked depletion of lymphocytes in the spleen and other tissues at necropsy are a hallmark of many severe bacterial and viral infections [48]. There is no evidence that filoviruses infect lymphocytes; instead, programmed cell death appears to be induced indirectly, possibly through the effects of factors such as TRAIL, FasL or nitric oxide released by infected macrophages [9,20,21,49,50]. It has been hypothesized that the massive loss of lymphocytes contributes to the severity of filovirus disease [9,21,25]. 

The only direct test of the importance of lymphocyte apoptosis in filovirus infection was performed by challenging transgenic or knockout mice deficient in various apoptotic pathways with maEBOV and assessing the occurrence of lymphocyte apoptosis and the course of illness [36]. Blocking the extrinsic apoptotic pathway by use of mice expressing a dominant negative form of FADD (utilized by TRAIL, Fas, TNF, *etc*.) in T cells reduced lymphocyte apoptosis after maEBOV infection. However, mice lacking Fas and TRAIL did not have decreased lymphocyte apoptosis, suggesting that multiple extrinsic signals induce apoptosis after infection. Interestingly, overexpression of Bcl-2 in white blood cells, which can block the intrinsic apoptotic pathway via inhibition of mitochondrial cytochrome-c release, also rescued lymphocytes from apoptosis after infection, suggesting that both intrinsic and extrinsic apoptotic pathways are utilized to induce lymphocyte apoptosis in maEBOV infection. However, inhibition of lymphocyte apoptosis did not result in improved mouse survival after maEBOV infection. Therefore, by exploiting the availability of transgenic mice, it was shown that lymphocyte apoptosis is not required for lethal EBOV infection. This result challenged and clarified earlier findings in the NHP model, underscoring the usefulness of the mouse model for understanding filovirus pathogenesis. 

### 4.5. Lymphocyte Responses to Filoviral Infection

Fatal filovirus infections of humans and NHPs are characterized by an initial decline in circulating lymphocytes, and correlates with increased lymphocyte apoptosis in blood, spleen, and lymph nodes during the course of infection [20,23,24,25,51]. These findings led to a hypothesis that lymphocyte apoptosis inhibited the generation of a successful immune response to filovirus infection [21,52], although this has been challenged as shown above [36]. The lymphopenia in EBOV-infected NHPs is followed by a rebound during the 1–2 days before death (Figure 2). This lymphocyte “spike” was observed in CD8^+^ and CD4^+^ T cells, B cells and NK cells [37]. Notably, lymphoblasts appear in the blood and lymphoid tissues of late-stage, lethally infected NHPs [21] and in humans infected with SUDV [53]. Further analysis of T cells from late-stage EBOV infection in NHPs revealed increased levels of the activation marker CD44 [37]. Similarly, lymphopenia and lymphocyte apoptosis are found after infection of mice with maEBOV or maRAVV [14,15,22], and lymphoblasts are found in maEBOV- and maRAVV-infected mice [15,19]. Also, increased levels of the activation marker CD44 in T cells have been found in maEBOV-infected mice, along with a decrease in CD127 and CD62L, indicative of activation [19].

The phenomena of late lymphocyte rebound and possible activation have been further studied in maEBOV-infected mice [19]. Splenocytes were isolated from moribund mice on day 7 postinfection and transferred to normal mice, which were then challenged with maEBOV. Animals that received splenocytes from infected mice survived, whereas controls that received cells from healthy donors died. CD8^+^ T cells purified from day 7 splenocytes were sufficient to transfer protection. The T cells showed a decrease in CD127 and CD62L, indicative of activation; they produced IFN-γ in response to EBOV peptides, but not to control MARV peptides. These data show that, despite massive lymphocyte apoptosis, maEBOV-infected mice are still able to develop a virus-specific CD8^+^ T-cell response, which may only fail to prevent death because tissue damage is already far advanced by the time the activated cells appear. A late increase in circulating lymphocytes is also observed in maRAVV-infected mice, and lymphoblasts are present in the spleen at necropsy [54].

Natural killer (NK) cells have also been shown to be important in protection against maEBOV. Mice vaccinated with a virus-like particle (VLP) vaccine 1–3 days before maEBOV infection required NK cells to protect against lethality; this protection was dependent on NK expression of perforin but not IFN-gamma [55].

Other studies have focused on the use of CD8^+^ and CD4^+^ T cell epitopes to track and quantitate immune responses to filovirus infection. Vaccination of mice with a vaccine expressing EBOV NP generated CD8^+^ T cells that were protective to naïve mice upon adoptive transfer. Epitope mapping revealed a 11-mer peptide that was the CD8^+^ T cell epitope [56]. Further investigation found multiple CD8^+^ T cell epitopes (11 in C57BL/6 mice and 9 in BALB/c mice) generated in mice vaccinated against maEBOV. In all cases but one, CD8^+^ T cells generated against these epitopes were protective upon adoptive transfer to mice infected with maEBOV. These epitopes have been shown to be a powerful tool to analyze immune responses in maEBOV-infected mice in a variety of studies from multiple investigators. Mice vaccinated with maEBOV VLP or inactivated maEBOV generated CD8^+^ T cell responses to the epitopes described above [57,58,59]. Similarly, maEBOV-specific CD8^+^ T cell responses were observed in mice that survived maEBOV infection after receiving antisense phosphorodiamidate morpholino oligomers [60]. Additionally, these epitopes were used to discover that CD8^+^ T cell responses against maEBOV are generated in lethal infection in mice [19]. Furthermore, tetramer staining to detect EBOV-specific CD8^+^ T cells has been used to analyze the magnitude and kinetics of CD8^+^ T cell responses in mice that survive or succumb to infection [61].

### 4.6. The Role of CD45 in Resistance to Infection

Following the discovery that compounds that inhibit the tyrosine phosphatase CD45 prevent apoptosis of macrophages infected with *Bacillus anthracis*, and that transgenic mice expressing low levels of CD45 are partially protected from anthrax infection *in vivo* [62], it was found that transgenic mice expressing low levels of CD45 were solidly resistant to maEBOV infection [63]. Resistance was dependent on the enzymatic activity of CD45, as knockout mice lacking the enzyme, mice expressing normal levels or transgenic mice expressing an enzymatically inactive mutant CD45 all succumbed to infection. CD8^+^ T cells and IFN-γ were required for protection in these mice, but CD4^+^ T cells and NK cells were dispensable [63]. Microarray studies suggested that several cellular pathways were differentially utilized during maEBOV infection and pointed to a possible role of IL-10 in pathogenesis. 

## 5. Vaccine Testing in Mouse Models

A major use of maEBOV since it was first developed has been the initial testing of candidate EBOV vaccines (Table 1). The models of lethal RAVV and MARV infection of immunocompetent mice are also being employed for vaccine evaluation. Strategies have included the use of noninfectious subunit vaccines and the development of viral vectors encoding the filoviral GP and other proteins (reviewed in [64]). 

### 5.1. Vaccines

A DNA vaccine encoding GP or NP completely protected mice from maEBOV challenge, although similar protection has not been reported in NHPs [65,66,67]. Enveloped EBOV virus-like particles (eVLP) containing GP and VP40 were generated in a mammalian expression system and used as vaccines [68,69]. BALB/c and C57Bl/6 mice were protected after eVLP vaccination (without adjuvant) in a dose-dependent manner from a range of challenge doses (~10–1,000 pfu or ~300–30,000 LD_50_) [70]. Adding QS-21 or RIBI adjuvant to the eVLP vaccine regimen completely protected guinea pigs from challenge after a single vaccine dose [71]. EBOV VLP vaccination of mice and guinea pigs prevented viremia and clinical symptoms through at least 28 days after EBOV challenge [70,71,72]. NHPs are also protected from EBOV, MARV, and RAVV infection after VLP vaccination [73,74]. Purified GP has also been shown to protect against maEBOV infection in mice [75]. While much of the work on developing filovirus vaccines has utilized virus vectors, it is clear from these studies that subunit vaccines have the potential to safely and specifically provide protection against lethal filovirus infection.

**Table 1:** Vaccines tested for protective efficacy against EBOV in animals and for immunogenicity in humans.

Vaccine	Mouse	Guinea Pig	NHP	Human	References
DNA	Yes	Yes	Partial	Immunogenic	[76,77,78]
VRP	Yes	Yes	Yes	NT	[76,79,80,81]
Adenovirus	Yes	Yes	Yes	Immunogenic	[74,82,83,84,85,86,87]
DNA/Adenovirus prime-boost	NT	Yes	Yes	NT	[77]
Virus-like particle	Yes	Yes	Yes	NT	[57,58,69,70,71,73,88,89]
Parainfluenza	NT	Yes	Yes	NT	[90,91,92]
VSV	Yes	Yes	Yes	NT	[93,94,95,96,97]
Vaccinia	NT	Yes	No	NT	[80,98]
Purified protein	Yes	Yes	Partial with DNA boost	NT	[75,76,79,99]
Inactivated virus	Mixed results (partial to complete protection)	Mixed results (partial to complete protection)	Mixed results (no to partial protection)	NT	[80,100,101]

NT, not tested.

A Venezuelan equine encephalitis (VEE) replicon particle (VRP) vaccine encoding EBOV GP was reported to be the most successful of the six structural proteins tested (GP, NP, VP24, VP30, VP35, and VP40) in mice, and VRP encoding MARV GP is sufficient to completely protect rodents and nonhuman primates against MARV [66,102,103,104,105]. This platform has also been reported to protect NHPs against EBOV [81].

Multiple groups have pursued the use of adenovirus-vectored vaccines for protection against filoviruses. Studies in mice compared immune responses of animals vaccinated with plasmids encoding EBOV GP, followed by boosting with adenovirus expressing EBOV GP, to those vaccinated with adenovirus expressing EBOV GP alone. This study found that the generation of anti-GP antibody responses to DNA prime/adenovirus boost was slower, but more protective than adenovirus alone [83,84]. Other investigations have shown the utility of both DNA prime/adenovirus boost or adenovirus vaccination alone in NHPs [74,77,85,106], and it should be noted that the DNA vaccine and adenovirus vaccine are the only filovirus vaccines to be tested for immunogenicity in humans to date. However, the question of the impact of pre-existing immunity to adenovirus has been raised regarding this vaccine platform. To address this issue, the mouse model was used to show that pre-existing immunity to adenovirus abrogated the efficacy of adenovirus-vectored EBOV vaccine given intramuscularly or orally, but not intra-nasally [82]. A live vesicular stomatitis virus (VSV) vaccine expressing GP was shown to protect mice from lethal EBOV infection when given pre- or post‑infection [107] and studies in guinea pigs and nonhuman primates have shown similar protection [93,94].

### 5.2. Mechanisms of Vaccine-Induced Immunity

Although the role of T cell responses in protection against filovirus infection is not well defined, both CD4^+^ and CD8^+^ T cells have been shown to be integral for achieving protection against filovirus infection [98,108,109]. For EBOV, several vaccine strategies, including liposomes encapsulating inactivated EBOV, DNA alone, DNA prime/adenovirus boost, VLPs, and VRP vaccines induce CD8^+^ T cell responses against EBOV GP and/or NP epitopes in mice [108,109,110,111,112]. The importance of CD8^+^ T cell responses was further demonstrated by protective adoptive transfer of NP-specific cells [108,113]. 

To examine the mechanisms of vaccine-induced protection, genetically modified mice were vaccinated with VLPs and then challenged with maEBOV. SCID mice, B cell-, CD8 T cell-, and IFN- γ-deficient mice vaccinated with eVLPs were not protected from lethal maEBOV challenge; in contrast, wild-type mice were completely protected by eVLP vaccination [57]. Therefore, both CD8^+^ T cell and antibody responses are important for protection against lethal maEBOV infection in this vaccination regimen. CD8^+^ T cell responses are thought to be important in survival in EBOV-infected humans [114]. However, it has been shown that CD8^+^ T cells may not be required for protection in VSV-EBOV-GP-vaccinated mice [94]. This study reinforces the theory that different filovirus vaccines or therapeutics likely act through different mechanisms [29,64].

### 5.3. Predictive Accuracy of Vaccine Testing in Mice

Are maEBOV- or maRAVV-infected mice accurate predictors of vaccine efficacy in NHPs? Some vaccine regimens that are protective in mice have failed to protect NHPs. For example, one study [80] used either inactivated EBOV, vaccinia virus expressing EBOV GP, or VRP expressing EBOV genes to vaccinate NHPs. These vaccines had shown protection in mice, but did not protect NHPs in this study from EBOV infection. However, as the authors noted, previous experiments had shown that inactivated preparations of EBOV had mixed results, with one study showing protection in baboons, and another failing to show protection in guinea pigs [101,115]. Additionally, subsequent efforts have shown that VRP vaccination can indeed protect macaques from EBOV infection [81]. Rather than diminishing the mouse model as a tool to test vaccines, these studies confirm the importance of the model to test, develop, and remodel vaccine candidates, as well as dissect mechanisms of immunity. No other model can be efficiently used for these purposes. It should also be noted that no filovirus vaccine that is efficacious in NHPs has failed to protect mice [64,65,70,74,94,103]. 

## 6. Antiviral Drug Testing

Mice have been used to evaluate antiviral drugs for filovirus infections since the early 1990s, when drugs were first tested in SCID mice infected with wild-type EBOV. However, the availability of maEBOV has greatly facilitated drug testing (Table 2). The recent development of murine models of lethal RAVV and MARV infection is also leading to their use for drug evaluation.

**Table 2:** Drugs and antibodies tested for protective efficacy against EBOV in various animal models.

Compound/Drug	Mouse	Guinea Pig	NHP	References
S-adenosylhomocysteine hydrolase inhibitors	Yes	NT	No	[116,117]
rIFN-αlpha2b	NT	NT	Delay to death	[43]
Convalescent blood	NT	NT	No	[118,119]
Monoclonal Antibodies	Yes ^1^	Yes	Partial ^2^	[56,120,121]
Polyclonal antibody/sera	Yes ^1^	Yes	Yes ^3^	[43,122,123,124,125,126,127]
PMO Antisense	Yes	Yes	Yes	[60,128,129]
siRNA	Yes	Yes	Yes	[130,131]
rNAPc2	NT	NT	3/9	[31,34]
rhAPC	NT	NT	2/11	[35]
FGI-103, 104, 106	Yes	NT	NT	[54,132,133]

^1^ Many (but not all) monoclonals and polyclonals tested protected mice from maEBOV; ^2^ KZ52 not protective; ch133 and ch226 cocktail protected 1/3 NHP; ^3^ Homologous immune IgG protected macaques when given 48 hours after infection; hyperimmune equine IgG protected baboons but not cynomolgous macaques; NT, not tested.

### 6.1. Passive Transfer of Antibodies or Immune Sera

Numerous experiments over the past two decades have attempted to determine whether antibody preparations can prevent or treat filoviral disease in rodent and NHP models. In one early study, 10 of 14 monoclonal antibodies protected mice from maEBOV infection, and some clones provided protection even when given 2 days after infection [134]. This and another study [121] showed that the relative ability of the different antibodies to neutralize plaque formation *in vitro* did not necessarily correlate with protection of the different antibodies *in vivo*, an important finding considering the lack of information regarding correlates of immunity in protection against filovirus infection. Additional studies demonstrated that in certain but not all instances, transfer of immune serum (or monoclonal antibodies) to naïve mice could provide protection from maEBOV infection [15,56,57,103,121,123,135,136].

Further tests in guinea pigs confirmed a possible use for antibody therapy against filoviruses. Equine IgG containing high levels of anti-EBOV antibodies have been shown to protect guinea pigs after guinea pig-adapted EBOV infection [43,137]. Passive transfer of sera from MARV convalescent animals or those vaccinated with the inactivated MARV preparations protected guinea pigs from challenge with the homologous strain in a dose-dependent manner, and at least partial protection of guinea pigs has been observed with administration of neutralizing monoclonal antibodies against MARV [138]. A monoclonal antibody (KZ52) derived from a human survivor of EBOV protected guinea pigs from guinea pig-adapted EBOV infection [127]. Other mouse-protective monoclonal antibodies, when give alone or in combination, either completely or partially protected (or extended the time to death of) guinea pigs from guinea-pig adapted EBOV [121,136]. 

Passive transfer of immune serum in NHPs has had an interesting history, with early reports of success of hyperimmune equine IgG against EBOV in baboons [139] that were not confirmed in a different NHP model (cynomolgous macaques) [124], leading to widespread speculation that passive transfer of antibodies was not an effective strategy. Additionally, some studies have reported *in vitro* evidence for antibody-dependant enhancement of infection [140,141]. Furthermore, KZ52, a monoclonal antibody that protected guinea pigs from EBOV infection, did not do so in NHPs [126]. Data in favor of a role for protective antibodies include the passive transfer success in NHPs [139] and possible protection in humans receiving whole blood from convalescent patients [119]. 

A recent study has put this matter to rest, showing complete protection against MARV or EBOV infection in NHPs with administration of specific IgG even at 48 hours after infection [125]. This is the most protective therapy for filoviruses ever published using NHP models. Importantly, this study used homologous IgG from immune animals to protect recipient NHPs, allowing for repeated dosing without inducing the anti-immunoglobulin antibodies that were found in other studies [124]. Therefore, passive transfer can protect not only mice and guinea pigs, but also NHPs from filovirus infection.

Additional studies following this report have shown success using monoclonal antibodies in NHPs. In one study, two monoclonal antibodies, previously shown to have some protection in rodent models, protected 1 of 3 NHPs from EBOV when administered prior to and after infection [142]. A second study reported 100% protection of EBOV-challenged NHPs given a cocktail of three neutralizing monoclonal antibodies against GP beginning 1 day after challenge; this same treatment was 50% effective when given 48 hours after challenge [143]. Together, these experiments suggest that both polyclonal and monoclonal antibody therapy can be effective in combating filovirus infection in NHPs.

### 6.2. Small-Molecule Drugs

Early studies to identify filovirus therapeutics focused on compounds that had already shown activity against other viruses. Several *S*-adenosylhomocysteine hydrolase (SAH) inhibitors were shown to be effective against maEBOV, perhaps by their ability to induce high quantities of type I IFN in infected mice [13,41,144]. While cyanovirin-A, a compound that binds to glycosylated proteins and inhibits HIV inhibition, could decrease maEBOV-induced cytopathic effects and delay the onset of the disease, it only extended the survival time of lethally challenged mice [145]. 

The non-peptidic small molecule FGI-103 has been shown to inhibit EBOV replication *in vitro.* Dosing in mice was performed to determine toxicity and pharmacokinetics of this compound. Subsequent studies showed that FGI-103 protected mice from maEBOV or maRAVV challenge, even when given as a single dose 24 hours after infection [133]. Treated mice exhibited reduced viral load, lower levels of MCP-1, TNF-alpha, IFN-gamma, and IL-6, and stable levels of liver enzymes in sera. 

Aman *et al.* demonstrated that a small-molecule compound designated FGI-106, administered one day after infection, can protect mice from lethal EBOV infection when used either in a prophylactic or therapeutic setting [54]. Interestingly, cell-based assays also identified inhibitory activity against divergent virus families including bunyaviruses (Rift Valley Fever Virus) and flaviviruses (dengue virus), which suggested that FGI-106 interferes with a common host pathway or viral mechanism utilized by different viruses [146].

### 6.3. siRNA and Antisense DNA

The use of RNA interference to treat viral infection has become an important therapeutic strategy (reviewed in [147]). The usefulness of the mouse model to test and refine antiviral treatments can be seen in the studies utilizing phosphorodiamidate morpholino oligomers (PMO) antisense. The efficacy of PMO targeting individual EBOV genes were tested in the maEBOV mouse model, and from these studies it was shown that the polymerase L alone was not an effective target for PMO treatment, although PMO against VP35, VP24, and L combined were effective in preventing lethality in mice. Therefore, subsequent studies combining PMO against these three genes were performed in NHPs, and this treatment improved survival after EBOV infection [60]. Additional studies used the maEBOV and maMARV mouse models to test and develop various chemically modified PMO molecules against a series of filovirus genes, resulting in PMO regimens which proved to be protective in NHPs when treatment started shortly after EBOV or MARV infection [148]. Earlier studies also used the maEBOV to screen modified PMOs for efficacy in maEBOV-infected mice [128,149]. These are pertinent examples of how filovirus mouse model platforms can be used as an inexpensive but effective testing ground for improved therapeutics.

siRNA has also been successfully used to treat filovirus infections. Guinea pigs [130] and NHPs [131] were protected from guinea pig-adapted EBOV when treated with liposome-encapsulated siRNA shortly after infection. Although mice were not used to evaluate efficacy against infection, they were used to test for toxicity in the absence of infection.

### 6.4. Interferon and Interferon Inducers

As mentioned above, SAH inhibitors that indirectly block the translation of viral mRNA by preventing cap methylation protect mice from maEBOV infection [116]. One of these inhibitors, 3-deazaneplanocin A, was chosen for further study. maEBOV-infected mice treated with the drug had increased type I IFN levels, and were completely protected from maEBOV infection; antibodies neutralizing type I IFN abrogated this protection [13,41]. IFN-alpha2b has been shown to prolong time-to-death and delay viremia in EBOV-infected NHPs [43].

### 6.5. Predictive Accuracy of Antiviral Drug and Antibody Testing in Mice

PMO antisense treatments that were generated and improved using maEBOV and maMARV models were also protective in NHP EBOV and MARV infections [60,148]. These studies suggest that filovirus mouse models can be important in refining drug treatments prior to experimentation in NHP models. However, not all therapeutic treatments that are effective in mice are equally effective in NHPs, although the antibody therapy discussed above reminds us that an initial unsuccessful result in NHPs does not necessarily rule out future success if treatments are modified. 

After intriguing early results suggesting possible protection with whole blood transfer in humans [119] or transfer of immune equine sera in NHPs [139], follow-up experiments suggested that equine sera transfer was not effective in NHPs [124,126]. Similarly, antibody or serum transfer protected mice from filoviruses in some studies but not in others [15,56,57,103,121,134]. 

Rather than representing a shortcoming of rodent models, the data suggested that antibody could protect, as long as the antibody quality, activity, species source, and administration were correct. For example, one of the early definitive NHP studies [124] found that hyperimmune equine IgG that had protected baboons from EBOV infection did not protect macaques from EBOV infection. However this study actually showed that viremia was completely suppressed as late as day 5 post-infection. Around this time, antibodies against equine immunoglobulins began to be generated, possibly abrogating the effectiveness of the treatment. This problem was solved 16 years later by using same‑species IgG (harvested from vaccinated, convalescent NHPs) to protect NHPs from EBOV or MARV infection when given 48 hours post-infection [125]. The lack of confirmation of the early success of serum transfer in NHPs was widely taken as a sign that passive transfer of antibody was not a viable therapeutic option, but something that could reliably protect only mice or guinea pigs. Indeed, some treatment regimens that protected mice did not protect guinea pigs or NHPs. However, in hindsight, antibody-mediated protection in mouse and guinea pig filovirus models was variable, just like in NHP models. The timing, route, dose, and type of antibody treatment were paramount in generating protection, which was confirmed in NHP studies. Importantly, the most effective antibody treatment in NHPs to date is transfer of homologous IgG, thereby suggesting that species-specific antibody may be the most effective therapy. Therefore, in many ways, mouse models kept alive the hope of antibody‑mediated protection that eventually became relevant (again) in NHP studies.

## 7. Strengths and Weaknesses of Murine Models

Filovirus mouse models have proven useful for studying basic aspects of replication, pathogenesis, and immune responses, and have also served as irreplaceable platforms for evaluating a wide range of vaccines, antibodies, antisense molecules and antiviral compounds. Immune responses, virus replication, and overall pathology (including liver dysfunction) appear quite similar to NHP models. The main differences seen in the maEBOV mouse model is a lack of fibrin deposition and the lethality of the virus only via the i.p. route. For maRAVV, the coagulopathy is more similar to NHP models but again, only i.p. infection is lethal. These differences should be kept in mind when conducting mouse experiments. However, the lack of data on the protective effects of anti-coagulants in filovirus-infected mice, and the variation in coagulopathy in different NHP species, suggests that more data need to be collected to understand the relevance of these issues.

It is not known why mice are resistant to mouse-adapted filoviruses when inoculated peripherally (s.c. or i.m.). CD8^+^ T cells and perforin, but not B cells or CD4^+^ T cells, are required for resistance to s.c. inoculation of ma-EBOV [45]. It is possible that the presence of Langerhans cells in the skin may have a role in contributing to protection from these viruses by activating CD8^+^ T cells, but at this point there are no data to prove this.

Mice are often used as a starting point for *in vivo* evaluation of vaccines or therapeutics against filoviruses. Successful treatments or vaccines against filovirus infections are then further tested in guinea pigs or hamsters, then finally NHPs. But is that a logical progression? 

While NHPs are the most accurate platform to model human filovirus infections, it is still not known whether they accurately predict the success of filovirus vaccines or therapeutics in humans. This is due in part to the scarcity of human data and the range of pathogenesis seen in different NHP species. Interestingly, many aspects of coagulopathy (such as fibrin deposition, clotting, and hemorrhage) in EBOV infection appears to vary according to which NHP model (cynomolgous macaque, rhesus macaque, grivets, African green monkey, marmosets, or baboons) and virus strain is used and which lab performs the experiment [6,150,151,152,153,154,155]. Other aspects of filovirus pathogenesis also appear to vary according to NHP species [150,152,155,156,157]. Functionally, these differences may be pertinent, as hyperimmune equine IgG was protective in baboons but not in macaques [124,139]. RESTV is highly pathogenic in most NHP (with the possible exception of African green monkeys [156]) but seems to be non-pathogenic in humans [158]. This suggests that a given NHP model may not be predictive for human filovirus infections and therapeutics, simply because there are no data of a successful treatment in any filovirus animal model also being effective in humans, and because there is very little human pathological data available for comparison. Of course, this is not to say that mice and guinea pigs are better or more predictive models, but rather that both rodent and NHP models are indispensible for studying filovirus pathogenesis and testing vaccines and therapeutics. Additional information in human infections and all filovirus models is needed in order to say how well a treatment in a given filovirus model transfers to human infections.

Guinea pigs are often used as a bridge between mouse and NHP studies. In EBOV infections, they have the advantage of having more fibrin deposition than mice [15,159]. In addition, they are similar to both mice and NHPs with regard to histopathological lesions and blood chemistry changes during filovirus infection. Furthermore, similar to NHPs, guinea pigs are susceptible to peripheral filovirus inoculation, unlike mice. However, there are few analytical reagents available for them. 

The recent development of a Syrian hamster model using maEBOV could be an important advance, especially given the apparent significant coagulopathy present in this model [160]. Testing maRAVV or maMARV for pathogenicity in hamsters would be an intriguing experiment.

The availability of gene knockout and transgenic mice make them unique compared to all other filovirus models for the ability to directly test the importance of certain genes in infection. In fact, this has been used in several studies to tease out genes important for successful vaccination or resistance to filovirus pathogenicity [13,32,36,45,57,61,63,123]. Also, the ability to perform adoptive transfer studies between individual mice is of great value [19,55,56,57,161]. There are considerably more antibodies and other reagents available for mice than for any other filovirus animal model for analysis of immunological responses, drug mechanisms of action, and viral pathogenesis.

### 7.1. Compartmentalization

Compartmentalization is a concept suggesting that animal models that accurately reflect human disease in one aspect can be useful even though they may not accurately reflect human disease in another aspect. For example, if the cytokine responses seen in baboon and mouse EBOV models resemble those in human infection, the data can be compared in both models as it relates to human disease. However, if mice have less coagulopathy than baboons, they are not instructive for that aspect of the disease, but are still informative for cytokine production studies. It must also be taken into account that one “compartment” that differs from human disease may affect another “compartment” in an unknown manner. For example, if mice lack the coagulopathy seen in EBOV-infected baboons, that might affect the production of a mouse cytokine that isn’t typically measured, resulting in an important unseen alteration of an informative (cytokine) compartment. In effect, compartmentalization is the approach that studies have taken, such as in immunological mouse filovirus experiments. In this manner, one can glean relevant information from all available animal models. It must be realized that at this point all filovirus models, are lacking important data to understand pathogenesis.

### 7.2. The Need for Adaptation of Filoviruses for Lethality in Mice

It is unknown why the different filoviruses appear to be diverse in their mouse adaptation, but it behooves us to look to the published literature. Mice lacking type I IFN are susceptible to certain filoviruses (EBOV, MARV), but not others (RESTV, TAFV) [13,39]. Different isolates of SUDV appear to vary in their lethality in type I IFN deficient mice [13,39]. Ebihara *et al.* [16] showed that mutations generated in the adaptation of EBOV to maEBOV that allowed the virus to better replicate in cells treated with type I IFN were absolutely required for generation of the lethal maEBOV. This finding suggesting the possible importance of type I IFN in EBOV infection is supported by the data showing that type I IFN deficient mice are susceptible to infection by wtEBOV [13,39]. However, wtEBOV and maEBOV VP24 appear to have similar inhibition of type I IFN signaling in mouse and human cells [162]; additional work needs to be done to verify the mechanisms of pathogenesis of maEBOV. Furthermore, wtRAVV VP40 inhibits type I IFN signaling in human cells but not mouse cells, while maRAVV VP40 inhibits type I IFN signaling in mouse cells [163], suggesting that different filoviruses use different proteins to affect innate immune responses. Together, these data remind us that filoviruses are functionally diverse in their ability to cause disease, and this is reflected in their differential pathogenesis in mouse strains. We have a tendency to group filoviruses as being similar in their mechanisms of pathogenesis, but even in humans, filoviruses are not equally pathogenic (notably RESTV, to a certain extent BGDV, and possibly TAFV and SUDV). Therefore, it is not surprising that adaptation to mice may require different modifications for different filoviruses.

## 8. Goals for Future Research

Many aspects of filoviral pathogenesis and treatment remain to be elucidated. For example, vaccination and therapeutic testing in the recent maRAVV and maMARV mouse models has only just begun. In addition, rather basic questions, such as which macrophage and dendritic cell subsets are infected and how their functions are affected *in vivo*, remain unanswered [164]. Further refinements of the mouse models may lead to novel discoveries about filovirus infections; indeed, the recent advances in humanized mouse models may provide a hitherto unavailable access to infected human cells in an *in vivo* setting [165]. 

### 8.1. Humanized Mice

Humanized mice are gaining in popularity, as many improvements in the generation and use of these mice have been made [166]. There are many types of humanized mice, but the two most commonly used are the Hu-PBL and Hu-SRC models. In the Hu-PBL model, immunodeficient mice (NOD/SCID/IL2Rgamma−/−, or NSG) are injected with human peripheral blood mononuclear cells. These mice engraft mostly human T cells, which proliferate as they generate an immune response to the immunodeficient mouse, resulting in graft-*versus* host disease that leads to death ~4–5 weeks after injection. However, this model can be very useful, as it generates a robust engraftment of functional human T cells in a cost-efficient manner, and has been used in the *in vivo* study of many infectious diseases, including the human immunodeficiency virus and dengue virus. Hu-PBL mice have been used to test whether human lymphocytes undergo apoptosis after EBOV infection *in vivo*. Interestingly, human lymphocytes underwent significant apoptosis after lethal maEBOV infection, but not after wtEBOV infection [30]. Additionally, there was an increase in several human cytokines in lethal maEBOV infection in these mice. 

The Hu-SRC model is based on the transplantation of human hematopoietic stem/progenitor cells in newborn NSG mice. These mice have normal life spans and engraft a wide range of human immune cells, including T cells, B cells, DCs, macrophages, and granulocytes. This model is very useful since it generates human cells that are thought, but not proven, to be important targets for filovirus replication (macrophages, DCs). Use of these models could help us understand filovirus replication and spread in human cells *in vivo*. HLA alleles have been correlated with protection against certain filoviruses in humans [47,53], so it would be interesting to test whether these alleles correlate with altered T cell responses *in vivo* in Hu-SRC mice engrafted with cells from different HLA types. 

### 8.2. Characterization of Intermediate-Passage Variants

A pertinent example of how to study which mutations are key for filovirus adaptation to different animals is the work by Ebihara *et al.* [16], in which reverse genetics was used to generate EBOV viral variants expressing certain mutations and then tested for pathogenesis in mice. Similar studies should be performed using strains of viruses that are generated at different stages of adaptation to mouse models.

The maEBOV was generated by 9 serial passages in progressively older mice [15], and maRAVV was made by serial passage in adult SCID mice to generate a SCID-adapted variant, followed by further passaging in adult immunocompetent mice [14,33]. Sequencing of different passages of maRAVV and maMARV (during both the SCID-adaptation and immunocompetent adaptation) revealed the order in which various mutations occurred [38]. Interestingly, the first mutation to occur in both viruses was in VP40, at the same site as the guinea-pig adapted EBOV, followed by a different VP40 mutation in both viruses [38]. The mutations found in maRAVV VP40 were shown to be important in the ability to block type I IFN signaling in mouse cells [163].

The use of intermediate passages of these viruses is a powerful tool to understand the sequential order and role of individual mutations to bring about lethal variants in different animal models. Other interesting questions could be answered by using intermediate passages of adapted filoviruses. Is the order of in which mutations occur important? Could mouse-or guinea pig-lethal MARV or RAVV variants be lethal without the consensus VP40 mutation? Are the UTR mutations relevant? The long‑term storage of each passage of virus in future adaptations of filoviruses to animal models would provide a wealth of information for future filovirus replication and pathogenesis studies. Often, these intermediate passage variants are lost over time due to turnover in personnel, increased institutional regulations on select agent storage, and limited BSL-4 freezer storage space. We propose that representative stocks of such intermediate passages be sent to one or more institutions for a repository of such variants.

## 9. Conclusion

The mouse models for filovirus infection have given the field excellent tools for studying pathogenesis, therapy, and vaccination for these difficult-to-study pathogens. Most vaccines and therapeutics have first been tested and refined in mouse models, saving time and expense for subsequent testing in NHPs. Basic research has also been made simpler by utilizing transgenic and knockout mice, which are not available in other filovirus models. Future research should be conducted to determine the similarity of mouse and other animal models to human filoviral infections.
